# Photoluminescence and photocatalytic properties of rhombohedral CuGaO_2_ nanoplates

**DOI:** 10.1038/srep21135

**Published:** 2016-02-18

**Authors:** Linlin Shi, Fei Wang, Yunpeng Wang, Dengkui Wang, Bin Zhao, Ligong Zhang, Dongxu Zhao, Dezhen Shen

**Affiliations:** 1 State Key Laboratory of Luminescence and Applications, Changchun Institute of Optics, Fine Mechanics and Physics, Chinese Academy of Sciences, No. 3888 Dongnanhu Road, Changchun, 130033, People’s Republic of China; 2 University of Chinese Academy of Sciences, Beijing, 100049, People’s Republic of China

## Abstract

Rhombohedral phase CuGaO_2_ nanoplates with a diameter of about 10 μm were synthesized via low temperature hydrothermal method. Room temperature and low temperature photoluminescence of the obtained CuGaO_2_ nanoplates were characterized. CuGaO_2_ nanoplates exhibited blue emission at room temperature and free exciton emission were appeared at low temperature. The blue emission is originated from defects such as Cu vacancies, which is the possible origin of p-type conductivity. The appearance of free exciton emission can demonstrate the direct bandgap transition behavior of CuGaO_2_ nanoplates. The as-prepared p-type CuGaO_2_ nanoplates were further decorated by n-type ZnO nanoparticles via calcination method to fabricate p-n junction nanocomposites. The nanocomposites exhibited enhanced photocatalytic activity which can be ascribed to the effective separation of photogenerated carriers by the internal electrostatic field in the p-n junction region, and the enhanced light absorption properties resulted from sub-bandgap absorption effect of p-n junction. This work has offered a new insight into the design of p-n junction devices using p-type CuGaO_2_ nanoplates.

Delafossite semiconductor CuMO_2_ (M = Al, Ga, In) has attracted much research interest in the past few decades because its intrinsic p-type conductivity has potential applications in constructing p-n junction devices^1^. CuMO_2_ has the fundamental character of indirect transition, however it has been demonstrated to have direct allowed transition occured at high energy side, therefore it can be classified as a wide band-gap semiconductor^2^,^3^. Since direct band gap is one of the most important features of semiconductor materials applied in photoelectric devices, delafossite semiconductors have promising prospects in optoelectronic applications^4^,^5^,^6^. However, studies of the optoelectronic properties of delafossite semiconductors have seldom been reported to date, mostly because of the intrinsic direct transitions are symmetry forbidden, and high quality delafossite semiconductors are difficult to obtain.

CuGaO_2_ follows the same rules in the delafossite families and has received considerable interests recently. Most efforts have been devoted to study structural, electronic and optical absorption properties of CuGaO_2_^7^,^8^, however just absorption measurement is not a sufficient determination method to study optical properties. To our knowledge, the reports based on the photoluminescence properties of CuGaO_2_ have not been published so far, and the application researches which have been reported were concentrated on transparent conductive film or p-type dye-sensitized solar cells^9^,^10^,^11^,^12^, there are few studies focused on the optoelectronic and photocatalytic applications.

It has been reported that the heteroepitaxial relationship at the interface between CuGaO_2_ and ZnO is highly probable because they have highly matched lattice parameters^4^, then the combination of CuGaO_2_ and ZnO in fabricating heterojunctions is an effective way to reduce the interface defects. Additionally, the n-type ZnO and p-type CuGaO_2_ can form a p-n junction with type II staggered band alignment. In a type II band alignment, the valence and conduction bands of CuGaO_2_ are higher than those of ZnO, which could thermodynamically facilitate the transfer of excited electrons and holes between them and subsequently enhance the separation of charge carriers to reduce their recombination^13^. Therefore the combination of CuGaO_2_ and ZnO is favorable for fabricating p-n junctions and have promising applications in photocatalytic activity.

In this work, CuGaO_2_ nanoplates were obtained through a simple hydrothermal method^14^,^15^, the obtained CuGaO_2_ nanoplates showed high crystalline and p-type properties. P-type properties of CuGaO_2_ nanoplates were examined by photoluminescence and electrical measurements. The blue emission was observed in CuGaO_2_ nanoplates, and p-type CuGaO_2_/n-type ZnO nanocomposite heterostructures were realized for applications in photocatalysis. To fabricate such CuGaO_2_/ZnO composite photocatalysts, we used a simple calcination reaction method in solution which can increase the contact areas between large size CuGaO_2_ nanoplates and small size ZnO nanoparticles. More importantly, the as-obtained products exhibited the enhanced photodegradation efficiency with respect to the individual constituents. The enhancement is attributed to the restraining recombination of photo-induced carries and the enhanced visible light absorption resulted from the formation of p-n junction. This work has offered new insight into the application of CuGaO_2_ materials and p-n junction based p-type CuGaO_2_ nanoplates.

## Results

### Structure analysis and photoluminescence properties of CuGaO_2_ nanoplates

**Figure 1:**
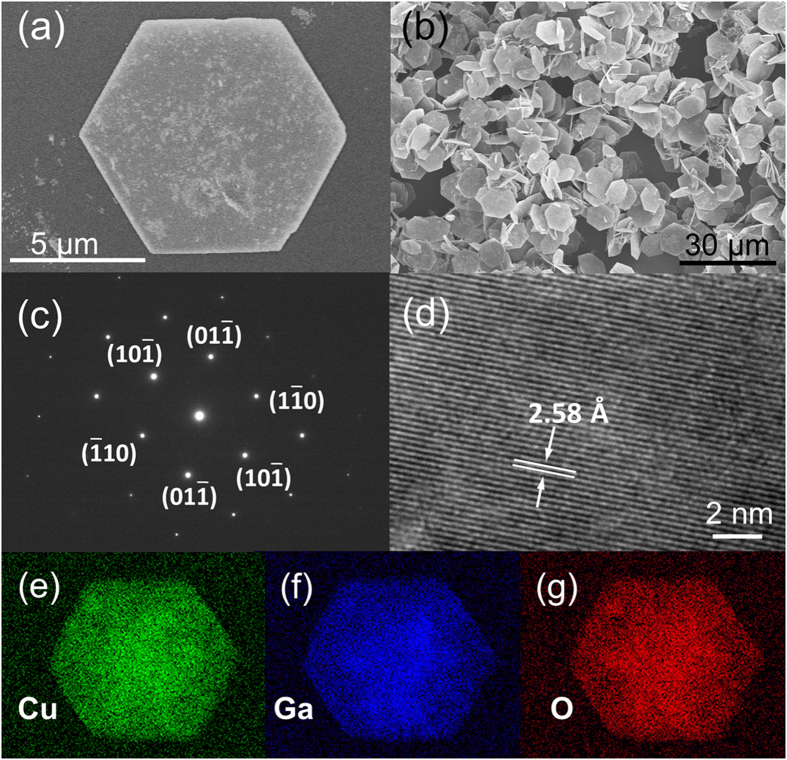
(**a**) SEM image of an individual CuGaO_2_ nanoplate, (**b**) Typical SEM image of multiple CuGaO_2_ nanoplates. The corresponding (**c**) SAED pattern and (**d**) HRTEM image taken from the nanoplates shows high quality crystallite. (**e**–**g**) Cu, Ga, and O elemental mappings, respectively.

The morphology and structure of CuGaO_2_ nanoplates were investigated by using the field-emission scanning electron microscopy (FESEM) and the transmission electron microscopy (TEM). As depicted in Fig. 1a, single CuGaO_2_ nanoplate exhibits hexagonal shape with an average diameter of about 10 μm. Figure 1b displays the SEM image of multiple CuGaO_2_ samples, which suggests the uniformity of the as-prepared CuGaO_2_ samples. Figure 1c shows the selected area electron diffraction (SAED) of CuGaO_2_, which confirms that CuGaO_2_ is well crystallized with a single phase. The corresponding high resolution FETEM image is further demonstrated to observe the fine structure of CuGaO_2_, the result is shown in Fig. 1d, the interlayer spacing of 2.58 Å calculated from TEM patterns confirm the proper phase formation of the material. Figure 1e–g show the elemental maps of individual CuGaO_2_ nanoplates. It is evident that Cu, Ga and O are homogeneously distributed in the nanoplates. The above results reveal that CuGaO_2_ nanoplates were highly crystallized.

**Figure 2:**
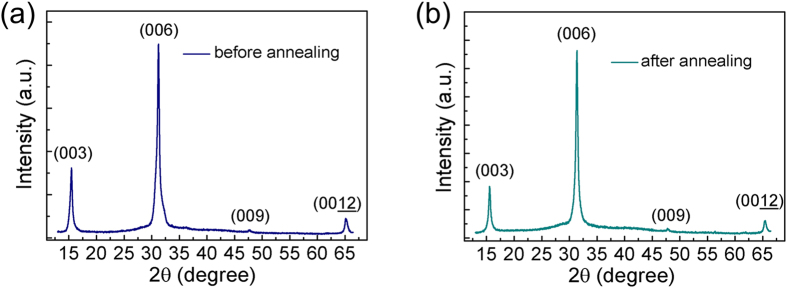
XRD patterns of the CuGaO_2_ nanoplates sample (**a**) before annealing and (**b**) after 400 °C annealing in air.

The X-ray diffraction (XRD) patterns of the CuGaO_2_ nanoplates are depicted in Fig. 2. CuGaO_2_ nanoplates have the rhombohedral delafossite crystal structure (JCPDS card No. 41-0255; space group a = 2.976 Å, c = 17.158 Å). In detail, the diffraction peaks located at about 15.52°, 31.19°, 47.72°, and 65.2°correspond to the (003), (006), (009), and (0012) planes of CuGaO_2_, respectively. No other peaks corresponding to Cu and Ga oxides impurities were observed in the pattern, suggesting the high purity of the as-synthesized products. To study the influence of the calcination temperature on the structure of CuGaO_2_ nanoplates, the XRD patterns of the CuGaO_2_ annealed under the reaction temperature of 400 °C in alcohol (solvent of the zinc acetate precursor and the temperature of ZnO synthesis) are showed in Fig. 2b. There is no change compared to the XRD patterns of untreated samples, indicating that CuGaO_2_ nanoplates are stable even after the calcination process under low annealing temperature.

**Figure 3:**
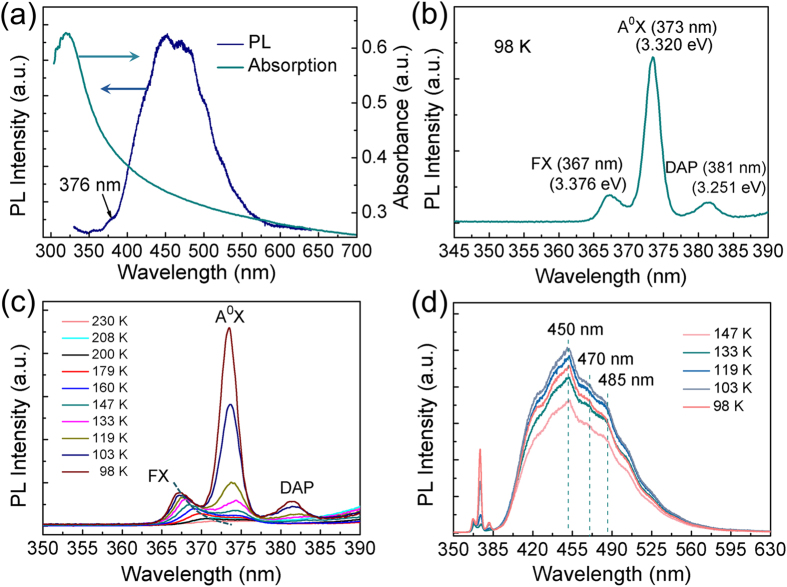
(**a**) Room temperature PL and absorption spectra of CuGaO_2_ nanoplates, (**b**) Low temperature PL spectrum of CuGaO_2_ nanoplates at 89 K, (**c**) Temperature-dependent PL spectra of CuGaO_2_ nanoplates at high energy side, (**d**)Temperature-dependent PL spectra of CuGaO_2_ nanoplates with broadband spectrum range.

The UV–Vis absorption spectrum of the as-prepared CuGaO_2_ nanoplates is shown in Fig. 3a. The corresponding optical band gap for direct allowed transition is estimated to be 3.53 eV (351 nm), which is in accordance with the theoretical calculation^2^,^3^,^16^. Room temperature photoluminescence (PL) spectrum is also shown in Fig. 3a, the small peak at 376 nm is attributed to near bandgap emission (NBE), which is much lower than the blue defect emission at room temperature.

In order to further confirm the direct band gap emission of CuGaO_2_ nanoplates, low temperature PL and temperature-dependent PL were performed. Figure 3b shows the low temperature PL spectra of CuGaO_2_ at 98 K, the occurrence of the peak at 367 nm (3.376 eV) can be attributed to the free exciton (FX) emissions of wide band-gap CuGaO_2_ nanoplates, the peak intensity becomes stronger as the temperature decreases. The peak concentrated at 373 nm (3.320 eV) and 381 nm (3.251 eV) are speculated to be the A^0^X and DAP emissions, respectively. It is well-known that the FX emission can be observed in the whole temperature range from 80 K to room temperature while the A^0^X emission decrease as the temperature increases. In Fig. 3c, A^0^X emission decreases extremely with increasing the temperature. Then it changes into FX emission and vanishes at 200 K because of the small localization energy. As the temperature increases, the A^0^X and DAP emissions become much weaker and disappear when the temperature is above 160 K, while the FX emissions exist even at room temperature with an apparent red-shift of peak position caused by the temperature increment. It is commonly presented and well-accepted that the emission of excitons localized by impurities dominates the PL spectra at low temperature, therefore the FX emissions can hardly be observed at low temperatures. From our experiments, the peak positions and temperature dependent behaviour of the free excitons at low temperature are firstly observed in CuGaO_2_, but have similarities compared with the other well-known wide band-gap semiconductors such as ZnO which reported in the literature^17^. Therefore, we attribute these two peaks to be A^0^X and FX emissions. These observations imply that the CuGaO_2_ nanoplates have direct transition character, which is considered to be favorable for optoelectronic applications. Because the properties of semiconductors are strongly affected and determinated by defects and impurities, the defect study is certainly worth to explain the mysterious of p-type conductivity in CuGaO_2_. Considering formation energy and transition energy level factors, some groups have calculated the relevant intrinsic defects including Cu-vacancy, O-interstitial, O-vacancy and Cu-interstitial in p-type CuGaO_2_ with the first-principle calculation methods. With the holes induced by Cu-vacancy and O-interstitial defects, the material present p-type conductivity^18^,^19^. Furthermore, some groups also deduced from theoretical calculation that Cu-vacancy is the most likely prominent defect that origins the p-type conductivity of CuGaO_2_^20^,^21^. The reports showed that all kinds of donor type intrinsic defects have high formation energy or have relatively deep transition energy levels, thus without the intentional doping, CuGaO_2_ present p-type conductivity. To date, on account of the luminescence properties of CuGaO_2_ has not been reported yet, we could conclude from CuAlO_2_ luminescence properties that the defect emission peak of CuGaO_2_ is more likely located in the range of 400–500 nm^22^. Temperature-dependent PL spectra of the defects emission are shown in Fig. 3d, a strong broad band emission in the range of 400–520 nm were observed at room temperature, the intensity of the blue defect emission is much stronger than that of NBE emission. Since all the emission peaks have no shift with the changing temperature, the mechanism of above emissions should be associated with the defects which act as trapped states within the band gap of CuGaO_2_. The Blue emission is composed of several peaks at 420 nm (2.95 eV), 450 nm (2.76 eV), 470 nm (2.64 eV), 485 nm (2.56 eV) and 520 nm (2.38 eV), respectively. Previous reports showed that the peak at 475 nm is originated from the Cu-vacancy defect energy level from theoretical calculation^20^. Moreover according to the above discussions, Cu-vacancy is the most likely defect which is the origin of p-type conductivity.

### Structure analysis and photoluminescence properties of of CuGaO_2_/ZnO nanocomposites

**Figure 4:**
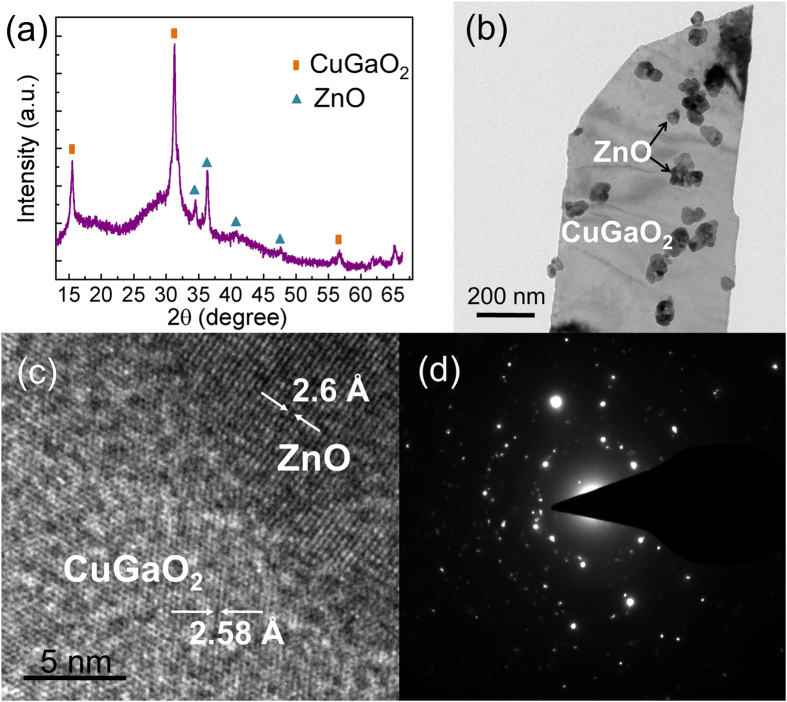
(**a**) XRD pattern of CuGaO_2_/ZnO nanocomposites, (**b**) TEM image of part of CuGaO_2_/ZnO nanocomposites. The corresponding (**c**) HRTEM image and (**d**) SAED pattern of CuGaO_2_/ZnO nanocomposites.

The XRD pattern of CuGaO_2_/ZnO nanocomposites made from annealing CuGaO_2_ nanoplates in zinc acetate precursor at 400 °C is depicted in Fig. 4a, both CuGaO_2_ phase and ZnO phase can be identified in the XRD pattern. The diffraction peaks of CuGaO_2_ phase of nanocomposites have no change compared with pure CuGaO_2_, the new emerging diffraction peaks centered at about 34.58°, 36.39°, and 47.61° correspond to the (100), (002), and (102) planes of wurtzite phase ZnO. The annealing process at 400 °C has not only formed ZnO nanoparticles but also removed the organic residues and enhanced the connection between the nanoplates and nanoparticles. Additionally, CuGaO_2_ nanoplates are stable when the thermal annealing temperature is performed under 400 °C in air according to the above discussion. Furthermore, it is observed that the peak intensity of nanocomposites is weaker than that of pure CuGaO_2_, which is due to the covering of ZnO nanoparticles.

After the attachment of tiny ZnO nanoparticles, the colour of CuGaO_2_ powder turned from black to dark grey. Further investigations of structure and morphology have been performed through TEM and HRTEM. As shown in Fig. 4b, the size of ZnO nanoparticles is about 50 nm, moreover, the formation of ZnO nanoparticles on the surface of CuGaO_2_ is evident from the TEM images. The HRTEM image of CuGaO_2_/ZnO composites in Fig. 4c shows that CuGaO_2_ and ZnO are both single crystalline, the dark area of tiny nanoparticles is ZnO, and the bright area is CuGaO_2_ nanoplate. Two distinct areas could be found with d spacing of 2.58 Å and 2.6 Å of CuGaO_2_ and ZnO, respectively, which presents very small mismatch between CuGaO_2_ and ZnO. Figure 4d shows the (selected are electron diffraction) SAED pattern of CuGaO_2_/ZnO nanocomposites, it is clear that the patterns are consist of ZnO rings and CuGaO_2_ lattice. The above results indicate the successful decoration of ZnO nanoparticles onto CuGaO_2_ nanoplates. The mixed phases may provide the interfaces to help the dissociation of electrons and holes when illuminated under visible light.

**Figure 5:**
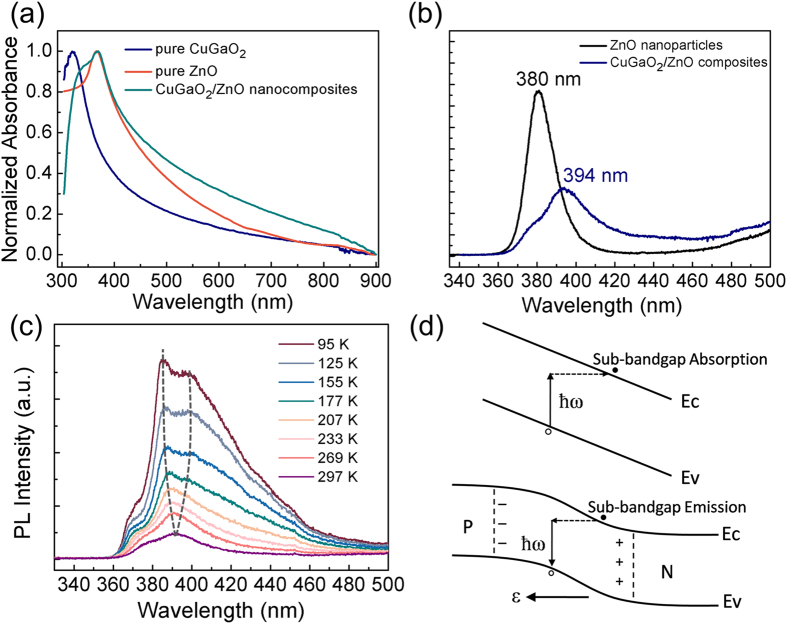
(**a**) The absorption spectra of the pure CuGaO_2_, ZnO and CuGaO_2_/ZnO composites, (**b**) The PL spectra of CuGaO_2_/ZnO composites at room temperature, (**c**) Temperature-dependent PL spectra of CuGaO_2_/ZnO composites, (**d**) The schematic diagram of sub-bandgap light absorption (up) and sub-bandgap light emission (down).

The absorption spectra of nanocomposites and pure CuGaO_2_ nanoplates are shown in Fig. 5a. After being decorated with tiny ZnO nanoparticles, a red shift of absorption peaks has been observed compared with the pure CuGaO_2_ nanoplates. There are some possible reasons responsible for this phenomenon: a) The delocalization of the electronic wave function resulted from the indirect spatial nature of excition and the large transition energy variation after the attachment of ZnO; b) The decreased potential well effected and extended photoresponse range caused by the relatively lower band gap of ZnO account for the red shift as well^23^. These above mentioned changes may have effects on on the electronic states and absorption properties.

To further investigate the optical properties of CuGaO_2_/ZnO p-n junction, the PL spectra of CuGaO_2_/ZnO nanocomposites and individual ZnO samples were measured at room temperature. Figure 5b shows that pure ZnO samples have a dominant peak centered at 380 nm, and the emission intensity of pure CuGaO_2_ is much weaker than ZnO at room temperature, interestingly, the CuGaO_2_/ZnO composites present a new dominant emission peak, which located at 394 nm. In addition, the emission intensity of ZnO has been suppressed. The above observation suggests an interpretation of the origin of new radiative recombination centers. Since the energy of the peak at 394 nm is smaller than the band-gap energies of either CuGaO_2_ or ZnO, which cannot be assigned to the transition of individual CuGaO_2_ or ZnO. At lower temperature, it can be observed from Fig. 5c that the peak at 394 nm splits into two emission peaks, these two peaks also shift with the temperature changing, indicates that 394 nm emission peak is affected by band-gap, not by the influence of defects. This result can be explained as the sub-bandgap absorption effect. This result might be explained by the sub-bandgap absorption effect which we discussed as follows.

If the concentration of the charged dopants is high enough and distribution layer of the p-n junction is narrow enough, the sub-bandgap light absorption could take place^24^, the schematic diagram is shown in Fig. 5d. It is well known that an intrinsic direct-gap semiconductor has no fundamental light absorption unless the energy of an incident photon ħω exceeds the bandgap E_g_. According to the Franz-Keldysh formula^24^, when the light frequency ω < E_g_/ħ, larger electric fields E will lead to stronger sub-bandgap absorption with the coefficient of


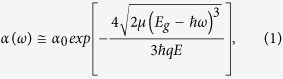


revealing an exponential tail deep in the forbidden gap. Here the pre-exponential factor α_0_ ~10^3^ to 10^4^ cm^−1^ is close to the fundamental bandgap edge light absorption coefficient in an intrinsic semiconductor in the absence of the electric field, q is the electron charge. The value


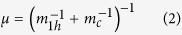


is a reduced effective mass of the conduction-band electron and light hole. If a build in electric field of the p-n junction is applied, the energy bands of a semiconductor are inclined along the direction of the field, so the electron in valence band can be promoted to the conduction band by absorbing a photon with energy ħω < E_g_, leaving a hole in the valence band. The similar absorption phenomenon has been observed in the previous literature, but the reasons remain undiscussed^25^. It is reported that the model of sub-bandgap absorption can also be applied to the fitting of room temperature PL^24^,^26^. When a p-n junction is established, the built-in internal electric field will lead to band bending, the p-n junction can emit the light which energy is smaller than the bandgaps of n-type and p-type materials. In addition, the PL intensity of the CuGaO_2_/ZnO composites is lower than the individual ZnO samples, which suggesting that the recombination of photogenerated electrons and holes could be suppressed by the formation of p-n nanojunctions. The suppression of carrier recombination could facilitate the performance enhancement of CuGaO_2_/ZnO nanocomposites in optoelectronic applications.

### Photocatalytic Activity

**Figure 6:**
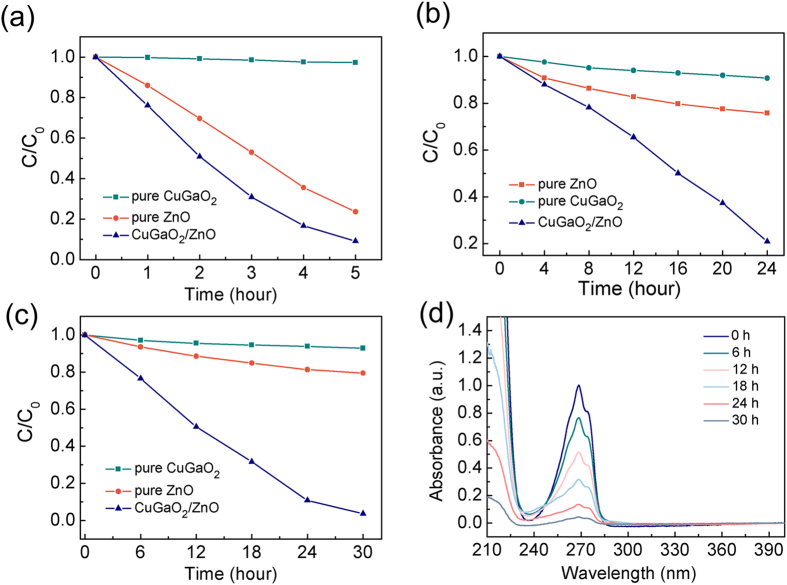
(**a**) Photocatalytic degradation curves of RhB using different photocatalysts under solar light irradiation, (**b**) Photocatalytic degradation curves of RhB using different photocatalysts under visible light irradiation, (**c**) Photocatalytic degradation curves of phenol using different photocatalysts under visible light irradiation, (**d**) UV-vis absorption spectra of phenol using different photocatalyst under visible light irradiation.

To develop the potential applications of CuGaO_2_/ZnO p-n junction nanocomposites, the photocatalytic performances of CuGaO_2_/ZnO nanocomposites compared with pure CuGaO_2_ nanoplates and ZnO nanoparticles were evaluated by degrading Rhodamine B (RhB) and phenol under solar/visible light. C/C_0_ stands for the degradation rate of organic dyes, where C is the concentration of RhB or phenol at each irradiated time, and C_0_ is the initial concentration when adsorption-desorption equilibrium is achieved. The photocatalytic activity comparisons of pure CuGaO_2_ nanoplates, pure ZnO nanoparticles and CuGaO_2_/ZnO composites were carried out by decomposing RhB under solar light irradiation. As shown in Fig. 6a, it is evident that the CuGaO_2_/ZnO composites exhibited higher photocatalytic activities than pure ZnO nanoparticles and CuGaO_2_ nanoplates. In order to further confirm the enhanced photodegradation capacity of CuGaO_2_/ZnO p-n junction composites, the photocatalytic activity of CuGaO_2_/ZnO composites were investigated through degrading RhB and phenol under visible light irradiation. In Fig. 6b, after 24 hours illumination, the concentration of residual RhB degraded by CuGaO_2_ and ZnO samples under visible light irradiation were still ~90% and ~80%, but the CuGaO_2_/ZnO samples present better degradation efficiency. Phenol is one of the toxic organic compounds that have no absorption of light over 350 nm, which can exclude the influence of photosensitization of the organic dyes under visible light irradiation. Figure 6c shows that CuGaO_2_/ZnO p–n junction samples display better degradation efficiency of phenol than pure CuGaO_2_ and ZnO samples under visible light illumination. Figure 6d shows the decreased absorption peak of phenol degraded CuGaO_2_/ZnO composites under visible light illumination, the decomposition of phenol has indeed taken place. The combination of CuGaO_2_ and ZnO has largely enhanced the visible light photocatalytic activity and phenol was degraded distinctly after 30 hours illumination. However, both pure ZnO and CuGaO_2_ samples had no obvious degradation reaction under visible light irradiation because they can only absorb ultraviolet light resulted from their large band gap energies. This result confirms that CuGaO_2_/ZnO p-n junction nanocomposites can greatly enhance the photocatalytic activity compared to the pure CuGaO_2_ or ZnO. The improved photocatalytic performance can be ascribed to the formation of the p-n junction. The transition of carriers has taken place between two materials, moreover, due to the sub-bandgap transition the nanocomposites can make use of the visible light with the energy band smaller than the band gap of two component materials.

## Discussion

**Figure 7:**
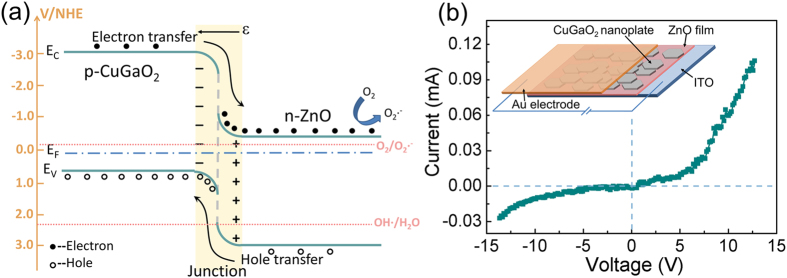
(**a**) Schematic diagram showing the energy band structure and electron-hole pair separation in the p-type CuGaO_2_/n-type ZnO, (**b**) I-V curve of p-type CuGaO_2_ nanoplates/n-type ZnO film shows rectification characters.

It has been reported that the design of p-n heterojunction with matching energy band potentials, greatly improves photocatalytic activity with visible-light irradiation^27^. This p-n heterojunction could effectively suppresses the photogenerated electron-hole recombination and promotes an interfacial electron transfer process^27^,^28^,^29^,^30^,^31^. The energy band structures of p-CuGaO_2_/n-ZnO at equilibrium is shown in Fig. 7a. CuGaO_2_ and ZnO are p-type and n-type wide band gap semiconductors, the wide band gaps of CuGaO_2_ and ZnO are about 3.56 eV and 3.37 eV, respectively. The energy band of CuGaO_2_ and ZnO match well with each other, the conduction band position of p-type CuGaO_2_ is much higher than that of n-type ZnO, their relative band positions make a type II staggered band alignment. This kind of alignment is beneficial for the separation of electron-hole pairs and the suppression of recombination^13^. When p-type CuGaO_2_ and n-type ZnO are contacted together, an inner electricfield from n-type ZnO to p-type CuGaO_2_ is thus established. Under light irradiation, both p-CuGaO_2_ and n-ZnO can be excited to generate electron-hole pairs, and then the photogenerated electrons on the conduction band of p-type CuGaO_2_ transfer to that of n-type ZnO, while photogenerated holes can migrate from the valence band of n-type ZnO to that of p-type CuGaO_2_ simultaneously. Such migrations of the photogenerated carriers can be attributed to the formation of internally formed electricfield. This electron-hole separation process has effectively increased the lifetime of holes on CuGaO_2_ surface, therefore the CuGaO_2_/ZnO heterojunction have higher photocatalytic activity than that of pure CuGaO_2_ and ZnO. Photocatalysis occurs when the semiconductor catalyst is exposed to the photons of energy equal to or higher than the band gap of the semiconductor under irradiation. The separated electrons in ZnO could react with the dissolved oxygen molecules to produce superoxide radicals (•O_2_^−^), which could generate the hydroperoxy, •HO_2_ radicals through protonation, finally produce hydroxyl radical •OH, which is a strong oxidizing agent for decomposing the organic dye^31^. Both •OH and •O_2_^−^ radicals are highly reactive and could effectively degrade organic pollutants like RhB and phenol^27^. Thus, an enhanced photocatalytic performance was observed on the p-CuGaO_2_/n-ZnO photocatalysts, compared with pure ZnO nanoparticals and CuGaO_2_ nanoplates. Moreover, because of the sub-bandgap absorption transition in p-n junction, p-CuGaO_2_/n-ZnO can absorb the light with the energy smaller than the two component materials, further enhance the photocatalytic performance.

To confirm that heterostructure of CuGaO_2_ nanoplates/ZnO could form a p-n junction diode, we construct a device composed of CuGaO_2_ nanoplates and ZnO film in order to test the electrical properties of CuGaO_2_/ZnO heterostructure, and schematically shown in the inset of Fig. 7b. Current–voltage (I–V) characteristics are depicted in Fig. 7b, the device shows good rectifying behaviour with a turn-on voltage of about 4 V. The result indicates that the CuGaO_2_ nanoplates have the characteristics of p-type conduction, and its combination with ZnO could form a typical p-n junction.

## Conslusion

In summary, a low temperature hydrothermal method has been used to synthesize CuGaO_2_ nanoplates. The blue emission and free exciton emission have been observed by room temperature and low temperature photoluminescence, indicating the existence of direct band-gap transition in CuGaO_2_ nanoplates. According to the I–V characteristics, CuGaO_2_ nanoplates exhibited inherent p-type conductivity property. CuGaO_2_ nanoplates were decorated with n-type ZnO nanopartitals to form CuGaO_2_/ZnO p-n junction nanocomposites, which exhibited enhanced photocatalytic activity of RhB and phenol under solar/visible light compared with pure CuGaO_2_ nanoplates and ZnO nanoparticals. This enhanced photocatalytic activity demonstrated the effective separation of carriers and the enhanced sub-bandgap absorption due to the formation of p-n junction. The study can provide a new insight into the design and fabrication of nanocomposites by using delafossite materials for optoelectronic applications.

## Method

### Preparation of CuGaO_2_ nanoplates

The recently developed hydrothermal method has provided chances to synthesize delafossite CuGaO_2_ at a relatively low temperature. The procedure was modified from the literature^14^. Briefly, 1 mmol Cu(NO_3_)_2_∙3H_2_O and 1 mmol Ga(NO_3_)_2_∙9H_2_O (stoichiometric mixture ratio of Cu/Ga = 1/1) were dissolved in deionized (DI) water, then 5 mmol KOH was added to co-precipitate Cu(OH)_2_ and Ga(OH)_3_ hydroxides. This prepared solution was diluted with ethylene glycol (EG) and DI water to fill up a total volume of 20 ml (capacity ratio of EG/DI water = 1/3). The final deep blue solution was put into a teflon bomb and placed in an oven with the growth temperature of 190 °C. After 56 hours of reaction time, the oven was switched off and the product was allowed to cool at room temperature. After appropriate washing and drying, the CuGaO_2_ nanoplates powder with black color were obtained.

### Preparation of CuGaO_2_/ZnO nanocomposites

For the heterostructure synthesis, the growth of ZnO nanoparticles was performed in a calcination way. The nanoplates were dispersed in the precursor ethanol solution (10 mM zinc nitrate) in a 25 ml glass bottle. The solution was then heated at 400 °C for 20 min to calcine the precursor. Then ZnO nanoparticles with the diameter of about 30 nm were obtained on the CuGaO_2_ nanoplates.

### Photocatalytic activity measurements

Photocatalytic activity of the prepared CuGaO_2_/ZnO samples was tested by decomposing RhB and phenol under solar/visible light irradiation. A 250 W xenon lamp with light intensity of 5 mW/cm^2^ was used as the light source, which has a glass filter to provide zero light intensity below 390 nm. The light absorption of the degraded solution was measured by the UV-3101 spectrophotometer. 1 mg pure ZnO nanoparticals, 1 mg pure CuGaO_2_ nanoplates and 1 mg ZnO/1 mg CuGaO_2_ nanocomposites was added respectively into 20 mL RhB or phenol solution in a glass bottle to produce a suspension for the degradation reaction at room temperature. The initial concentration of RhB and phenol are 10 mg/L and 50 mg/L, respectively. Before light irradiation, the suspension was stirred in dark for 30 min to ensure an adsorption-desorption equilibrium of RhB and phenol on the surface of the photocatalyst. The degradation reaction process was monitored by measuring the concentration of RhB and phenol as a function of irradiation time through UV-vis absorption spectra.

### Characterization

Field-emission scanning electron microscopy (FESEM, Hitachi S-4800), and high-resolution transmission electron microscopic (TEM, JEOL JEM-2100F) were used to study the surface morphology and microstructure of the samples. The absorption spectra were carried out using a Shimadzu UV-3101 PC spectrophotometer. Photoluminescence (PL) measurement was carried out with a JY-630 micro-Raman spectrometer employing the 325 nm line of a He-Cd laser as the excitation source. Low temperature PL measurements were acquired using the 325 nm line of a He-Cd laser and analyzed using a one meter grating spectrometer (SPEX 1704) equipped with a photomultiplier tube (Hamamatsu model R3310-02) cooled to 80 K. The current-voltage characteristics of the p-type CuGaO_2_ nanoplates/n-type ZnO film device were measured using a Keithley 2611A measurement system. The device was fabricated by assembling n-ZnO film (synthesized by sol-gel method on ITO substrate) with CuGaO_2_ nanoplates film (thermal evaporation of Au electrode on Si substrate followed by dropping CuGaO_2_ nanoplates).

## Additional Information

**How to cite this article**: Shi, L. *et al.* Photoluminescence and photocatalytic properties of rhombohedral CuGaO_2_ nanoplates. *Sci. Rep.* **6**, 21135; doi: 10.1038/srep21135 (2016).

## References

[b1] Kawazoe H. *et al.* P-type electrical conduction in transparent thin films of CuAlO_2_. Nature 389, 939–942 (1997).

[b2] Pellicer-Porres J. *et al.* On the band gap of CuAlO_2_ delafossite. Appl. Phys. Lett. 88, 181904 (2006).

[b3] Kumar M., Zhao H. & Persson C. Study of band-structure, optical properties and native defects in A^I^B^III^O_2_ (A^I^=Cu or Ag, B^III^=Al, Ga or In) delafossites. Semicond. Sci. Technol. 28, 065003 (2013).

[b4] Forticaux A., Hacialioglu S., DeGrave J. P., Dziedzic R. & Jin S. Three-dimensional mesoscale heterostructures of ZnO nanowire arrays epitaxially grown on CuGaO_2_ nanoplates as individual diodes. ACS Nano 9, 8224–8232 (2013). 23952783 10.1021/nn4037078

[b5] Ling B. *et al.* Color tunable light-emitting diodes based on p^+^-Si/p-CuAlO_2_/n-ZnO nanorod array heterojunctions. Appl. Phys. Lett. 97, 013101 (2010).

[b6] Wang J. *et al.* Solution synthesized p-type copper gallium oxide nanoplates as hole transport layer for organic photovoltaic devices. J. Phys. Chem. Lett. 6, 1071−1075 (2015). 26262872 10.1021/acs.jpclett.5b00236

[b7] Liu Q. J., Liu Z. T., Chen J. C., Feng L. P. & Tian H. First-principles study of structural, mechanical, electronic and optical properties of 3R- and 2H-CuGaO_2_. Physica B 406, 3377–3382 (2011).

[b8] Han M. J. *et al.* Temperature dependent phonon evolutions and optical properties of highly c-axis oriented CuGaO_2_ semiconductor films grown by the sol-gel method. Appl. Phys. Lett. 99, 131104 (2011).

[b9] Yu M. Z., Draskovic T. I. & Wu Y. Y. Cu(I)-based delafossite compounds as photocathodes in p-type dye-sensitized solar cells. Phys. Chem. Chem. Phys. 16, 5026–5033 (2014). 24477758 10.1039/c3cp55457k

[b10] Yu M. Z., Natu G., Ji Z. Q. & Wu Y. Y. P-type dye-sensitized solar cells based on delafossite CuGaO_2_ nanoplates with saturation photovoltages exceeding 460 mV. J. Phys. Chem. Lett. 3, 1074–1078 (2012). 26288038 10.1021/jz3003603

[b11] Xu Z. *et al.* Remarkable photocurrent of p-type dye-sensitized solar cell achieved by size controlled CuGaO_2_ nanoplates. J. Mater. Chem. A 2, 2968–2976 (2014).

[b12] Renaud A. L. *et al.* Impact of Mg doping on performances of CuGaO_2_ based p-type dye-sensitized solar cells. J. Phys. Chem. C 118, 54–59 (2014).

[b13] Marschall R. Semiconductor composites: Strategies for enhancing charge carrier separation to improve photocatalytic activity. Adv. Funct. Mater. 24, 2421–2440 (2014).

[b14] Srinivasan R. *et al.* Tuning the size and color of the p-type wide band gap delafossite semiconductor CuGaO_2_ with ethylene glycol assisted hydrothermal synthesis. J. Mater. Chem. 18, 5647–5653 (2008).

[b15] Yu M. Z., Draskovic T. I. & Wu Y. Y. Understanding the crystallization mechanism of delafossite CuGaO_2_ for controlled hydrothermal synthesis of nanoparticles and nanoplates. Inorg. Chem. 53, 5845–5851 (2014). 24832380 10.1021/ic500747x

[b16] Gillen R. & Robertson J. Band structure calculations of CuAlO_2_, CuGaO_2_, CuInO_2_, and CuCrO_2_ by screened exchange. Phys. Rev. B 84, 035125 (2011).

[b17] Wu K. W., He H. P., Lu Y. F., Huang J. Y. & Ye Z. Z. Dominant free exciton emission in ZnO nanorods. Nanoscale 4, 1701–1705 (2012). 22293981 10.1039/c2nr11773h

[b18] Nie X. l., Wei S. H. & Zhang S. B. Bipolar doping and band-gap anomalies in delafossite transparent conductive oxides. Phys. Rev. L 88, 066405 (2002). 10.1103/PhysRevLett.88.066405 11863832

[b19] Scanlon D. O. & Watson G. W. Conductivity limits in CuAlO_2_ from screened-hybrid density functional theory. J. Phys. Chem. Lett. 1, 3195–3199 (2010).

[b20] Fang M. *et al.* Optical properties of p-type CuAlO_2_ thin film grown by rf magnetron sputtering. Applied Surface Science 257, 8330–8333 (2011).

[b21] Tate J. *et al.* Origin of p-type conduction in single-crystal CuAlO_2_. Phys. Rev. B 80, 165206 (2009).

[b22] Byrne D., Cowley A., Bennett N. & McGlynn E. The luminescent properties of CuAlO_2_. J. Mater. Chem. C 2, 7859–7868 (2014).

[b23] Xu X. J. *et al.* Controlled growth from ZnS nanoparticles to ZnS–CdS nanoparticle hybrids with enhanced photoactivity. Adv. Funct. Mater. 25, 445–454 (2015).

[b24] Foygel M., Brenden E., Seguin J. H. & Osipov V. V. Sub-bandgap optical electro-absorption in the field of a P-N junction. phys. stat. sol.(b) 203, 255–263 (1997).

[b25] Li X. N. *et al.* A templated method to Bi_2_WO_6_ hollow microspheres and their conversion to double-shell Bi_2_O_3_/Bi_2_WO_6_ hollow microspheres with improved photocatalytic performance. Inorg. Chem. 51, 6245–6250 (2012). 22591138 10.1021/ic300454q

[b26] Katahara J. K. & Hillhouse H. W. Quasi-fermi level splitting and sub-bandgap absorptivity from semiconductor photoluminescence. J. Appl. Phys. 116, 173504 (2014).

[b27] Liu L. M., Yang W. Y., Li Q., Gao S. A. & Shang J. K. Synthesis of Cu_2_O nanospheres decorated with TiO_2_ nanoislands, their enhanced photoactivity and stability under visible Light illumination, and their post-illumination catalytic memory. Appl. Mater. Interfaces 6, 5629–5639 (2014). 10.1021/am500131b 24673595

[b28] Lin J. J. *et al.* Nano-p-n junctions on surface-coarsened TiO_2_ nanobelts with enhanced photocatalytic activity. J. Mater. Chem. 21, 5106–5113 (2011).

[b29] Lu M. X. *et al.* p-MoO_3_ nanostructures/n-TiO_2_ nanofiber heterojunctions: controlled fabrication and enhanced photocatalytic properties. Appl. Mater. Interfaces 6, 9004–9012 (2014). 10.1021/am5021155 24869636

[b30] Tian Q. Y. *et al.* Tube-like ternary αFe_2_O_3_@SnO_2_@Cu_2_O Sandwich heterostructures: synthesis and enhanced photocatalytic properties. Appl. Mater. Interfaces 6, 13088–13097 (2014). 10.1021/am5029439 24991983

[b31] Peng Y. *et al.* Novel one-dimensional Bi_2_O_3_–Bi_2_WO_6_ p–n hierarchical heterojunction with enhanced photocatalytic activity. J. Mater. Chem. A 2, 8517–8624 (2014).

